# Venous thromboembolism in patients undergoing surgery for lung cancer: a *post hoc* analysis of the Cancer-VTE Registry

**DOI:** 10.1093/jjco/hyae090

**Published:** 2024-07-22

**Authors:** Riken Kawachi, Tetsuya Okano, Nobuyasu Awano, Masaru Matsumoto, Jun Hosokawa, Atsushi Takita, Mari S Oba, Hideo Kunitoh

**Affiliations:** Department of Respiratory Surgery, Nihon University School of Medicine, 30-1 Oyaguchi-kamicho, Itabashi-ku, Tokyo 173-8610, Japan; Department of Respiratory Medicine, Nippon Medical School Chiba Hokusoh Hospital, 1715 Kamagari, Inzai-shi, Chiba 270-1694, Japan; Department of Respiratory Medicine, Japanese Red Cross Medical Center, 4-1-22 Hiroo, Shibuya-ku, Tokyo 150-8935, Japan; Department of Pulmonary Medicine and Oncology, Graduate School of Medicine, Nippon Medical School, 1-1-5 Sendagi, Bunkyo-ku, Tokyo 113-8603, Japan; Primary Medical Science Department, Daiichi Sankyo Co., Ltd., 3-5-1 Nihonbashi-honcho, Chuo-ku, Tokyo 103-8246, Japan; Data Intelligence Department, Daiichi Sankyo Co., Ltd., 1-2-58 Hiromachi, Shinagawa-ku, Tokyo 140-8710, Japan; Department of Medical Statistics, Toho University, 5-21-16 Omorinishi, Ota-ku,Tokyo 143-8540, Japan; Department of Clinical Data Science, Clinical Research & Education Promotion Division, National Center of Neurology and Psychiatry, 4-1-1 Ogawa-higashi-cho, Kodaira-shi, Tokyo 187-8551, Japan; Department of Medical Oncology, Japanese Red Cross Medical Center, 4-1-22 Hiroo, Shibuya-ku, Tokyo 150-8935, Japan

**Keywords:** lung cancer, surgery, venous thromboembolism, chemotherapy, D-dimer

## Abstract

The relationship between lung cancer surgery and venous thromboembolism (VTE) in Japan has not been elucidated. This was a *post hoc* analysis of the Cancer-VTE Registry. The 1057 patients who underwent surgery for lung cancer were divided into the surgery alone (SA) group (*n* = 598) and the surgery plus chemotherapy (SC) group (*n* = 459), and the 1-year incidences of VTE and cerebral ischemia were analyzed. In the SA and SC groups, composite VTE was observed in one (0.2%) and 15 (3.3%) patients, respectively, and cerebral ischemia was observed in eight (1.3%) and four (0.9%) patients, respectively. Lymph node metastasis was more common in patients with D-dimer >1.2 μg/ml (odds ratio: 1.781, *P* = .004). SA had a low risk of VTE but a high risk of cerebral ischemia. Chemotherapy increases the risk of VTE. The D-dimer level was related to VTE and advanced cancer.

## Teaser

There is a lower incidence of venous thromboembolism in Japanese patients who undergo only surgical treatment for lung cancer than in those who undergo surgery plus chemotherapy.

## Introduction

Venous thromboembolism (VTE) is a hypercoagulable condition comprising deep vein thrombosis and pulmonary embolism. VTE is a common and serious complication of surgery [1]. However, no large-scale prospective studies have evaluated VTE in patients with cancer in Japan, and the relationship between cancer type and VTE, and risk factors has not been fully clarified. Therefore, we created the Cancer-VTE Registry, a multicenter prospective registry study of Japanese patients [2].

The incidence of VTE after surgery for lung cancer is reported to be between 1.6% and 17.9%, but varies widely among publications [1]. The frequency of VTE varies depending on the patient risk factors, surgical technique, and other factors [3]. In patients undergoing surgery for lung cancer, the risk of developing VTE is high immediately after lung resection, and then slowly decreases [3,4]. The timing of VTE onset in patients undergoing surgery and chemotherapy is important for determining when prevention and monitoring are needed.

This study aimed to clarify the incidence of VTE and the timing of thrombotic events in patients who underwent surgery for lung cancer. Our secondary objective was to assess the role of D-dimer levels in predicting advanced cancer and prognosis.

## Patients and methods

The Cancer-VTE Registry (UMIN000024942) is a nationwide, multicenter, observational, prospective cohort analysis of the incidence of VTE in patients with six cancer types in Japan. The methods of the Cancer-VTE Registry have been previously described in detail [2]. The ethics committee or institutional review board at each participating institution approved the study protocol (approval number: RK-170613-5; approval date: 16 June 2016). The study was conducted in accordance with the Declaration of Helsinki and the Ethical Guidelines for Medical Science Studies on Human Subjects by the Japanese Ministry of Education, Culture, Sports, Science and Technology and the Ministry of Health, Labour and Welfare. All participants provided written informed consent at the time of registration.

This study was a *post hoc* analysis of the Cancer-VTE Registry [5,6]. A total of 2377 patients with lung cancer were enrolled in the Cancer-VTE Registry, of whom 1057 patients underwent surgery and were included in the present study. Patients were divided into two groups: the surgery alone (SA) group that received SA or surgery plus radiotherapy, and the surgery plus chemotherapy (SC) group that received SC with or without radiotherapy. Patients were classified into these two groups to clarify the incidence of VTE by surgical treatment alone. In the present study, symptomatic VTE and incidental VTE requiring treatment were defined as composite VTE, while cerebral infarction, transient ischemic attack, and systemic embolic events were defined as cerebral ischemia. Composite VTE, hemorrhage, cerebral ischemia, and all-cause death were analyzed in both groups. The relationship between D-dimer levels and lymph node metastasis was also analyzed.

Categorical variables were presented as *n* (%), while continuous variables were presented as mean, standard deviation, and median. Incidences were calculated as population proportions at the 1-year follow-up. The 95% confidence intervals (CIs) for the incidences were calculated. Odds ratios and 95% CIs were calculated to determine the relationship between D-dimer levels and lymph node metastasis. All statistical analyses were conducted using SAS software (version 9.4; SAS Institute Inc., Cary, NC, USA).

## Results

### Patient characteristics

The patient characteristics are shown in Table 1. The mean age was 68.8 years, and 69.6% of the patients were male. The SA group included more older adult patients than the SC group. Descriptive comparisons showed no differences between the SA and SC groups in physical characteristics, prevalence of hypertension, or smoking history. Fewer patients with a history of VTE, cerebral infarction, or intracranial hemorrhage received chemotherapy. There were no differences between the SA and SC groups in the surgical approach (thoracoscopy versus open thoracotomy). D-dimer levels were higher in the SA group than the SC group.

**Table 1:** Patients’ characteristics

		**Treatment**	
	**Total cohort**	**Surgery alone (SA)**	**Surgery plus chemotherapy (SC)**
Number of patients	1057	598	459
Males, *n* (%)	736 (69.6)	422 (70.6)	314 (68.4)
Age years, mean ± SD (median)	68.8 ± 9.4 (70.0)	71.5 ± 8.6 (73.0)	65.4 ± 9.4 (67.0)
Age over 65 years, *n* (%)	770 (72.8)	488 (81.6)	282 (61.4)
BMI < 25 kg/m^2^, *n* (%)	791 (74.8)	444 (74.2)	347 (75.6)
BMI ≥ 25 kg/m^2^, *n* (%)	264 (25.0)	153 (25.6)	111 (24.2)
**Smoking status**			
Current smoker, *n* (%)	81 (7.7)	45 (7.5)	36 (7.8)
Previous smoker, *n* (%)	724 (68.5)	409 (68.4)	315 (68.6)
Never smoked, *n* (%)	252 (23.8)	144 (24.1)	108 (23.5)
**Performance status (ECOG)**			
0, *n* (%)	908 (85.9)	512 (85.6)	396 (86.3)
1, *n* (%)	137 (13.0)	79 (13.2)	58 (12.6)
2, *n* (%)	12 (1.1)	7 (1.2)	5 (1.1)
**Presence of complications**			
Hypertension	466 (44.1)	284 (47.5)	182 (39.7)
Atrial fibrillation	35 (3.3)	24 (4.0)	11 (2.4)
Liver dysfunction	15 (1.4)	9 (1.5)	6 (1.3)
Peptic ulcer	25 (2.4)	19 (3.2)	6 (1.3)
**Medical history**			
VTE	6 (0.6)	6 (1.0)	0 (0.0)
Cerebral infarction	46 (4.4)	34 (5.7)	12 (2.6)
Intracranial hemorrhage	16 (1.5)	12 (2.0)	4 (0.9)
Gastrointestinal bleeding	11 (1.0)	7 (1.2)	4 (0.9)
Bed rest for 4 days or more	4 (0.4)	3 (0.5)	1 (0.2)
Oral anticoagulant therapy, *n* (%)	45 (4.3)	28 (4.7)	17 (3.7)
Creatinine clearance ≤50 ml/min, *n* (%)	136 (12.9)	106 (17.7)	30 (6.5)
D-dimer level in μg/ml, mean ± SD (median)	0.94 ± 1.99 (0.60)	1.07 ± 2.43 (0.60)	0.77 ± 1.15 (0.50)
D-dimer level >1.2 μg/ml, *n* (%)	112 (10.6)	79 (13.2)	33 (7.2)
Platelet count, ≥350 × 10^9^/L, *n* (%)	87 (8.2)	36 (6.0)	51 (11.1)
Hb < 10 g/dL, n (%)	14 (1.3)	10 (1.7)	4 (0.9)
WBC count >11 × 10^9^/L, *n* (%)	39 (3.7)	20 (3.3)	19 (4.1)
**Cancer subtype**			
SCLC, *n* (%)	11 (1.0)	3 (0.5)	8 (1.7)
Non-SCLC, *n* (%)	972 (92.0)	557 (93.1)	415 (90.4)
Adenocarcinoma, *n* (%)	578 (54.7)	304 (50.8)	274 (59.7)
Squamous cell carcinoma, *n* (%)	279 (26.4)	180 (30.1)	99 (21.6)
NOS, *n* (%)	115 (10.9)	73 (12.2)	42 (9.2)
Others, *n* (%)	74 (7.0)	38 (6.4)	36 (7.8)
Lymph node metastasis, *n* (%)	360 (34.1)	171 (28.6)	189 (41.2)
Distant metastasis, *n* (%)	36 (3.4)	13 (2.2)	23 (5.0)
**Cancer stage**			
IB, *n* (%)	397 (37.6)	257 (43.0)	140 (30.5)
II, *n* (%)	410 (38.8)	234 (39.1)	176 (38.3)
III, *n* (%)	215 (20.3)	94 (15.7)	121 (26.4)
IV, *n* (%)	35 (3.3)	13 (2.2)	22 (4.8)
**Type of surgery**			
Thoracotomy, *n* (%)	609 (57.6)	368 (61.5)	241 (52.5)
VATS, *n* (%)	441 (41.7)	232 (38.8)	209 (45.5)

BMI, body mass index; VTE, venous thromboembolism; SCLC, small cell lung cancer; SD, standard deviation; NOS, not otherwise specified; VATS, video-assisted thoracic surgery.

### Incidences of composite VTE, cerebral ischemia, hemorrhage, and all-cause death

Composite VTE developed in 16 patients (1.5%, 95%CI: 0.9–2.4) in the total cohort. The incidence of composite VTE was 0.2% (95% CI: 0.0–0.9) in the SA group and 3.3% (95% CI: 1.8–5.3) in the SC group. Cerebral ischemia and hemorrhage were more frequent in the SA group than the SC group, with SA group incidences of 1.3% (95% CI: 0.6–2.6) and 1.0% (95% CI: 0.4–2.2), respectively. There was no difference between the two groups in all-cause of death. Patients with advanced disease stages more frequently developed composite VTE (1.8%, 95% CI: 0.9–3.2) and cerebral ischemia (1.7%, 95% CI: 0.8–3.0) (Table 2).

**Table 2:** Incidences of composite VTE, cerebral ischemia, hemorrhage, and all-cause death during follow-up

			**Incidence of events during follow-up**							
			**Composite VTE**		**Cerebral ischemia**		**Hemorrhage**		**All-cause death**	
		***n*, %**	**Patients with events, *n* (%)**	**95% CI**	**Patients with events, *n* (%)**	**95% CI**	**Patients with events, *n* (%)**	**95% CI**	**Patients with events, *n* (%)**	**95% CI**
	Total cohort	1057 (100)	16 (1.5)	0.9–2.4	12 (1.1)	0.6–2.0	7 (0.7)	0.3–1.4	76 (7.2)	5.7–8.9
**Cancer treatment**	Surgery alone	598 (56.6)	1 (0.2)	0.0–0.9	8 (1.3)	0.6–2.6	6 (1.0)	0.4–2.2	43 (7.2)	5.3–9.6
	Surgery and chemotherapy	459 (43.4)	15 (3.3)	1.8–5.3	4 (0.9)	0.2–2.2	1 (0.2)	0.0–1.2	33 (7.2)	5.0–9.9
**Surgical procedure**	Thoracotomy	609 (57.6)	7 (1.1)	0.5–2.4	6 (1.0)	0.4–2.1	6 (1.0)	0.4–2.1	45 (7.4)	5.4–9.8
	VATS	441 (41.7)	7 (1.6)	0.6–3.2	5 (1.1)	0.4–2.6	1 (0.2)	0.0–1.3	25 (5.7)	3.7–8.3
**VTE at baseline**	With VTE	22 (2.1)	2 (9.1)	1.1–29.2	0 (0)	0.0–15.4	0 (0)	0.0–15.4	3 (13.6)	2.9–34.9
	Without VTE	1035 (97.9)	14 (1.4)	0.7–2.3	12 (1.2)	0.6–2.0	7 (0.7)	0.3–1.4	73 (7.1)	5.6–8.8
**Stage**	IB	397 (37.6)	4 (1.0)	0.3–2.6	1 (0.3)	0.0–1.4	4 (1.0)	0.3–2.6	14 (3.5)	1.9–5.8
	II, III, IV	660 (62.4)	12 (1.8)	0.9–3.2	11 (1.7)	0.8–3.0	3 (0.5)	0.1–1.3	62 (9.4)	7.3–11.9
**D-dimer level**	≤1.2 μg/ml	911 (86.2)	11 (1.2)	0.6–2.2	11 (1.2)	0.6–2.2	5 (0.5)	0.2–1.3	59 (6.5)	5.0–8.3
	>1.2 μg/ml	112 (10.6)	2 (1.8)	0.2–6.3	1 (0.9)	0.0–4.9	2 (1.8)	0.2–6.3	16 (14.3)	8.4–22.2

Composite VTE includes symptomatic VTE and incidental VTE requiring treatment.

Cerebral ischemia includes cerebral infarction, transient ischemic attack, and systemic embolic event.

VTE, venous thromboembolism; VATS, video-assisted thoracic surgery.

### Timing of composite VTE and cerebral ischemia after surgery

In the SA group, only one patient developed composite VTE in the first postoperative week. In the SC group, composite VTE developed in three patients (0.7%) in the first postoperative month, three (0.7%) in the second month, two (0.4%) in the third month, and seven (1.5%) in the fourth month or later. Cerebral ischemia developed in 12 patients (1.1%), of which 41.7% developed within 2 weeks postoperatively. Cerebral ischemia also developed in five patients (41.7%; 3 in the SA group and 2 in the SC group) in the fourth postoperative month or later.

### Relationship between D-dimer level and lymph node metastasis

Of the 1057 patients, 360 (34.1%) had lymph node metastases. Lymph node metastases were significantly more frequent in the high D-dimer level (>1.2 μg/ml) group (45.5%, 95% CI: 36.1–55.2) than the normal D-dimer level (≤1.2 μg/ml) group (31.9%, 95% CI: 28.9–35.1). The odds ratio was 1.781 (95% CI: 1.198–2.650, *P* = .004).

## Discussion

This study provides clinical information and real-world data on the incidence of VTE in patients with lung cancer who underwent surgery during a 1-year follow-up after VTE screening in Japan. The incidences of composite VTE were 1.5% in the total cohort and 0.2% and 3.3%, respectively, in the SA and SC groups, which were much lower than the reported rates [1]. The reasons for the low VTE incidence could not be determined but may have been related to the fact that 4.3% of the patients were treated with direct oral anticoagulants. Furthermore, direct oral anticoagulants were initiated in patients in whom VTE was detected at screening, which may have reduced the incidence of VTE during follow-up. Various chemotherapeutic agents have been reported to increase the risk of VTE [7]. However, in the present study, despite differences in surgical techniques, the addition of chemotherapy increased the incidence of composite VTE by 3.1%.

The timing of composite VTE was similar to previous reports, with a higher incidence in the immediate postoperative period, followed by a gradual decline, with a total of 56.3% of patients developing VTE within 3 months. Although the incidence of VTE was increased immediately after surgery, it continued to occur thereafter, albeit in smaller numbers. A meta-analysis examining the incidence of VTE in relation to the postoperative period showed that the incidence of VTE decreased over time, with 47.1% of VTE events occurring in the first postoperative week, 26.9% in the second postoperative week, 15.8% in the third postoperative week, and 10.1% in the fourth postoperative week [4]. The risk of VTE is highest immediately after surgery and declines thereafter, but it has also been reported that the risk of VTE declines after 3 months but increases again after 6 months, especially in patients with stage IV lung cancer [3]. Thus, physicians should be mindful that VTE can occur at any time after surgery. Regular follow-up with patients is crucial. Additionally, patients should be informed of the risk and be attentive to symptoms, such as lower extremity edema.

Cerebral ischemia must also be considered in patients undergoing pulmonary surgery. Cerebral infarction is more likely to occur after pulmonary surgery [8]. In addition to the patient’s condition and comorbidities, the risk of cerebral infarction is increased immediately after surgery due to the transection of the pulmonary veins. Thrombus formation is thought to be caused by paroxysmal atrial fibrillation due to altered blood flow in the left atrium and turbulence formation in the long leftover pulmonary veins [8]. Among the patients who developed cerebral ischemia, 41.7% of cases occurred within 2 weeks after surgery, suggesting a direct association with the surgical procedure. Therefore, rather than solely monitoring for the occurrence of cerebral ischemia, it is necessary to minimize the length of pulmonary vein stumps during surgical procedures.

Another finding of the present study was that patients with high D-dimer levels had a more advanced cancer stage and a poorer prognosis than those with normal D-dimer levels. Recently, several studies have investigated the relationship between D-dimer levels and prognosis in patients with lung cancer [9]. Activation of the blood coagulation system is reportedly associated with the invasive and migratory biological behavior of tumors [10]. However, the underlying mechanism by which elevated D-dimer levels are associated with decreased survival in patients with lung cancer remains unclear.

The present study has several limitations. First, most patients undergoing surgery for lung cancer in Japan do not routinely receive heparin postoperatively, and mechanical prophylaxis is the only primary prophylaxis; however, these factors were not analyzed. Second, certain details of the surgical procedure, including mediastinal lymph node dissection, were not available. Therefore, although the incidence of VTE varies in accordance with the surgical procedure, this association could not be analyzed because the surgical procedures were not registered. Third, information on the timing of chemotherapy and surgery, such as preoperative chemotherapy, salvage surgery, conversion surgery, adjuvant chemotherapy, and chemoradiation, was unavailable.

In conclusion, VTE is less common in patients undergoing SA for lung cancer, but cerebral ischemia can occur independently of chemotherapy and should be carefully monitored. VTE can occur long after surgery for lung cancer and should be monitored over time. Patients with lung cancer with elevated D-dimer levels should be monitored for cancer progression, as they are more likely to have advanced disease than patients with normal D-dimer levels.

## Data Availability

The anonymized data underlying the results presented in this manuscript may be made available to researchers upon submission of a reasonable request to the corresponding author. The decision to disclose the data will be made by the corresponding author and the funder, Daiichi Sankyo Co., Ltd. The data disclosure can be requested for 36 months from the article publication.
